# Comparative Study of Y-Split Recession versus Faden Technique for Management of Infantile Esotropia in Egyptians

**DOI:** 10.1155/2018/3408614

**Published:** 2018-08-01

**Authors:** Nermeen Badawi, Ahmed Taha Ismail

**Affiliations:** ^1^Ophthalmology Department, Faculty of Medicine, Menoufiya University, Shebin El-Kom, Menoufiya, Egypt; ^2^Ophthalmology Department, Faculty of Medicine, Ain-Shams University, Abbaseya, Cairo, Egypt

## Abstract

**Purpose:**

This study compares the results of Y-split recession versus de Decker's (modified Cüppers) Faden techniques of medial rectus (MR) muscles for the management of essential infantile esotropia (IET).

**Patients and Methods:**

Fifty patients had IET divided into Group A who underwent Y-split recession of MR muscles and Group B who underwent de Decker's Faden technique of MR muscles. All patients had complete ophthalmic examination done including deviation angle measurement and met the inclusion criteria of the study. Operations were performed using general anesthesia. Patients were followed up at day 1, week 1, and months 1, 3, and 6 after operation.

**Results:**

The mean age distribution for group A was 21.56 months (SD 12.55) and for group B was 21.4 months (SD 12.35), and the mean postoperative follow-up interval was 6 months for both groups. The preoperative maximum angle of deviation in both groups ranged from 15 to 40 degrees, while the minimum angle of deviation ranged from 10 to 20 degrees. Immediately postoperatively both groups showed 88% of patients with satisfactory results (within 10 degrees of orthotropia). Group A showed two patients (8%) with ET and one patient (4%) with exotropia (XT). For group B, it showed one patient (4%) with ET and two patients (8%) with XT. Three patients in each group underwent a second intervention. All patients remained within the satisfactory range.

**Conclusion:**

The results of this study suggest that both techniques show comparable results for the correction of IET.

## 1. Introduction

Infantile esotropia can be managed by minimizing torque (*T*) of medial rectus (MR) muscles. *T* = *F* ∗ *r* [1], where *F* represents the force exerted by MR and *r* represents the lever arm. *T* can be reduced by minimizing *F* or *r* [1–6].

Techniques for *r* reduction include Faden operation, a concept introduced by Cüppers [7–11], which decreases MR *r* by suturing it to back of the globe. Many studies showed that this controls strabismus [12–14].

Y-split recession mostly involves MR splitting and resuturing thus reducing MR *r* and effective muscle *F* [15–19]. It is a concept introduced by Priglinger 1990 and published 1994. Long-term studies and statistical analysis of the outcome after surgical therapy were followed by Haslwanter et al. [18] and Hoerantner et al. [19].

This study aims at comparing both techniques of IET.

## 2. Patients and Methods

The fifty patients involved in this study with big-angle infantile ET were divided into Group A (Y-split recession of MR) and Group B (de Decker's Faden technique who, in contrary to Cüppers, preferred to leave the muscle in place and secure it with a triple loop to prevent its sliding through the suture). All patients were subjected to complete ophthalmic examination and met the inclusion criteria, which were (1) ET diagnosed by an ophthalmologist before the age of sixth months, (2) absence of fixation preference, and (3) ET maximum angle values varying from 15 to 40 degrees and were determined using Hirschberg, Krimsky, or prism alternate cover test for distance and near (whenever possible with proper conversion) with optimal refractive correction (whenever needed). Due to variability of strabismus angles, maximum and minimum (static) angles as well as the difference between both (dynamic) were measured at each distance three times, and the average was taken for each distance. Exclusion criteria were neurological abnormalities, developmental delays, nystagmus, anomalous head posture (AHP), convergence excess, or ocular structural abnormalities. Cycloplegic refraction was performed, and glasses were used in patients with hyperopia of +3.0 diopter (D) or more for at least one month to exclude any accommodative component. Duction and version movements, oblique muscle overaction, pattern (V or A), and dissociated strabismus were recorded. All operations were performed under general anesthesia using the operating microscope.

Follow-up visits were conducted at 1 day; 1 week; and 1, 3, and 6 months postoperatively. Fixation assessment in young children and “illiterate E game” and Snellen acuity chart testing were performed in elderly children for vision assessment. AHP, deviation angle (static and dynamic), convergence, and ocular motility were assessed during follow-up examinations. Rates of reoperation for residual ET or consecutive XT were determined.

Before surgeries, all legal representatives of the patients were told the expected complications. All of them signed informed consents matching Helsinki Declaration.

### 2.1. Group A: Y-Split Recession Surgical Technique

**Figure 1:**
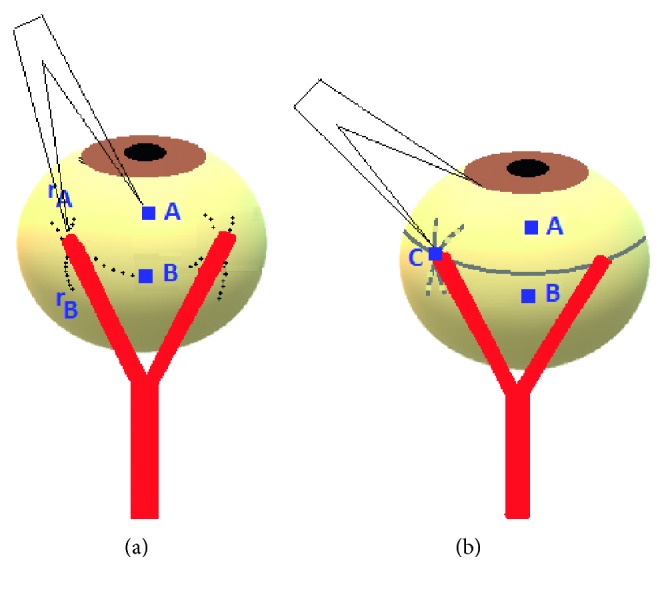
A sketch of Y-split recession, side view. (a) The first orientation point (“A”) is given by the middle of the natural muscle insertion. The second orientation point (“B”) is located 6 mm straight behind A. With a compass, the distance *r*_A_ is marked with color on the globe. The same procedure is repeated from B, with the distance *r*_B_. The intersection of the two marked lines indicates the new insertion points for the split-muscle halves. (b) The “control distance” (“C”) ensures correct placement of the new insertion points.

For the Y-split recession, as described by Priglinger and Hamenter [17], Haslwanter et al. [18], and Hoerantner et al. [19], the surgical parameters were chosen such that *T* reduction was about 26%. The surgeon split MR muscles for a length of 15 mm and sutured the two split-halves to the sclera using nonabsorbable sutures. The two split-halves formed an angle of 62.8 ± 5.7 degrees. The muscle was, first, split for a distance of 15 mm, to detect the new accurate insertion for the split-muscle halves, and surgeon performed the following: the first point, labeled “A,” was located in the middle of the original insertion. The second point, labeled “B,” was located 6 mm straight behind A. With the compass centered at A, the distance “*r*_A_” was marked on the globe with methylene blue (Figure 1) [19]. Similarly with the compass centered at B, the distance “*r*_B_” was marked on the globe. The crossing points of the methylene blue lines locate the new insertion of the split-muscle halves. Both muscle halves were secured using 5/0 polyester suture with a spatulated needle (Ethicon®).

### 2.2. Group B: de Decker's Faden Technique

**Figure 2:**
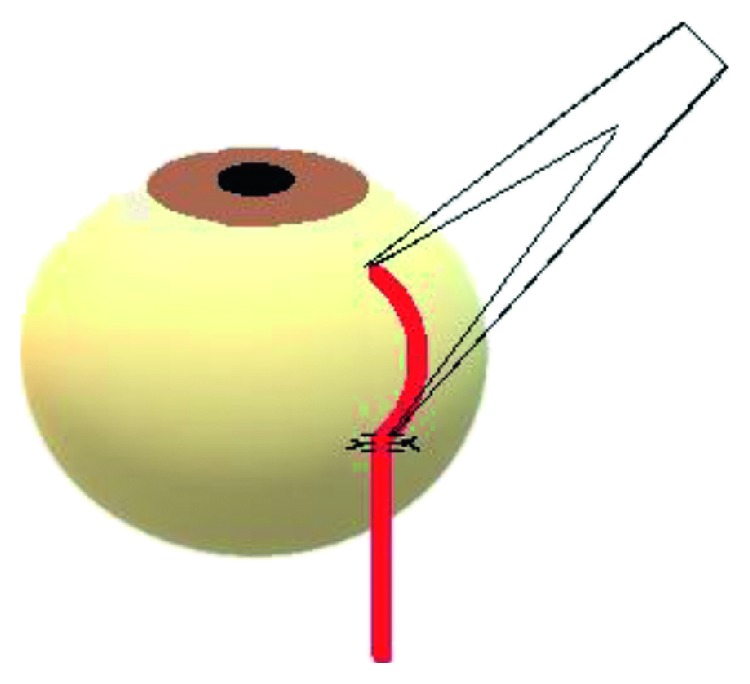
A sketch of de Decker's Faden technique, side view.

All children received a simple retroequatorial strapping of both MR 14 mm posteriorly from their scleral insertion with a nonabsorbable 5/0 polyester suture with a spatulated needle (Ethicon). This was performed after careful dissection of connective tissues around the muscle body as far as the site of suturing the muscle. Two sutures were used at the muscle edge. The sutures were simply fixed to the sclera on both sides of the muscle fixing its margins in a triple loop fashion to prevent its sliding through the suture (Figure 2).

No orthoptic exercises were performed after surgery. Patients continued to wear glasses (if needed) and were watched regularly for alignment and change of refraction. Statistical analysis was done using mean analysis, correlation *t*-tests, Mann–Whitney test, and Friedman test. A *P* value lower than 0.05 was considered statistically significant.

## 3. Results

**Figure 3:**
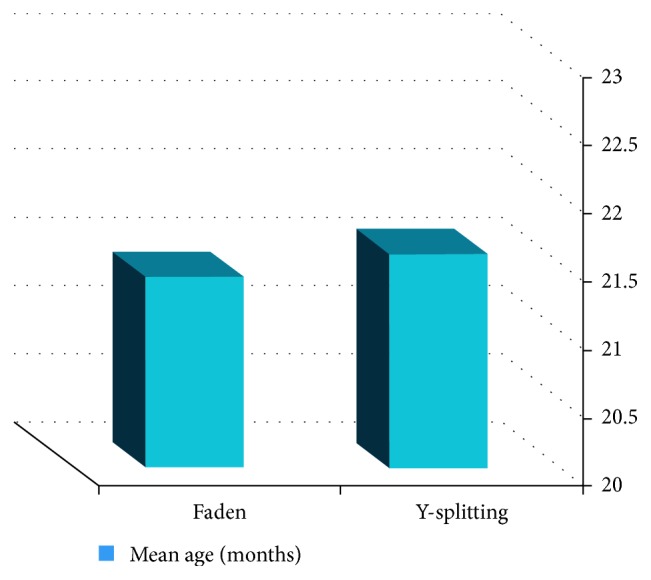
Patients' profile showing patients' distribution by age.

The age distribution for Group A (Y-split recession) ranged from 9 to 54 months, with a mean of 21.56 months (SD 12.55) and for Group B (de Decker's Faden technique) ranged from 8 to 48 months, with a mean of 21.4 months (SD 12.35). There was a statistically nonsignificant difference in age distribution between both groups by the Mann–Whitney test. This is shown by patients' distribution by age (Figure 3).

The mean postoperative follow-up interval was 6 months for both groups. The mean surgical time for Group A (Y-split recession) was 50 minutes (SD 13.50) and for Group B de Decker's Faden technique was 40 minutes (SD 10.05) that means that Group A consuming more operative time by 20% than Group B. It should be noted that the technique of Y-split recession included more steps and necessitated that the limbal incision has to be steeper up to the intended refixation points and MR has to be split about 15 mm from insertion. Yet, the surgical area was more anterior and hence safer. No patient developed nystagmus, AHP, or convergence excess. The average values of the maximum, minimum (static), and dynamic strabismus angles preoperatively, one day; one week; and one , three, and six months postoperatively are shown in (Tables 1–3).

**Figure 4:**
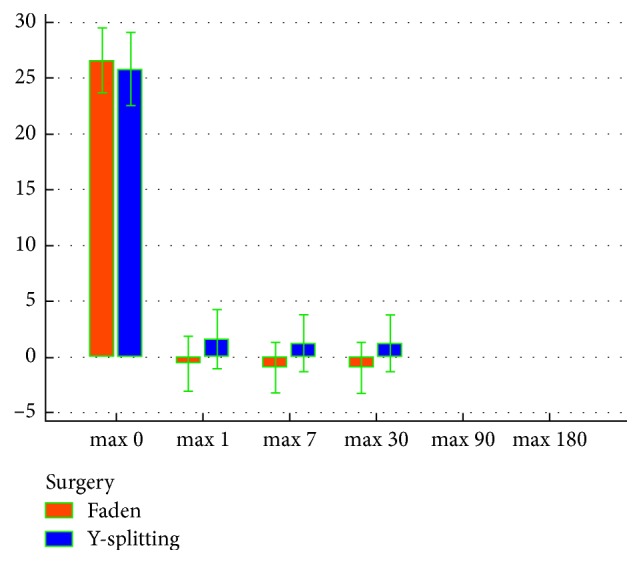
Preoperative and postoperative maximum strabismus angles in both groups.

**Figure 5:**
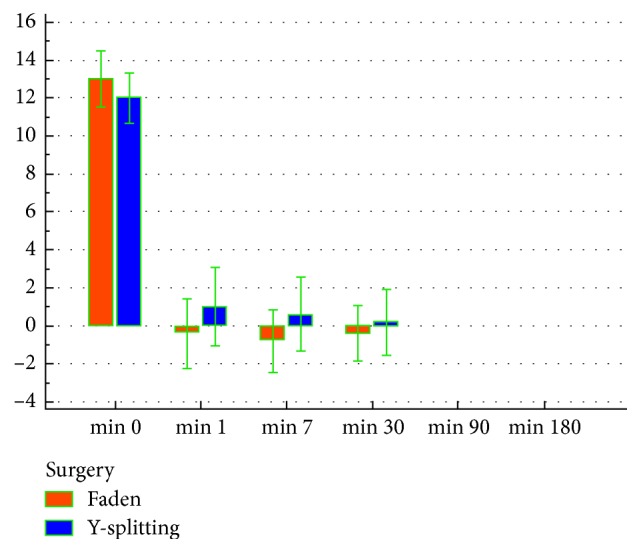
Preoperative and postoperative minimum strabismus angles in both groups.

The mean postoperative follow-up interval was 6 months for both groups. Criteria of patients satisfaction were postulated as (i) satisfactory surgical outcome within 10 degrees of orthotropia and (ii) unsatisfactory surgical outcome if ET or XT ≥ 10 degrees. Both groups showed a statistically highly significant reduction in postoperative maximum, minimum (static), and dynamic strabismus angles compared to preoperative ones in every postoperative follow-up visit by the Friedman test (*P* < 0.001). However, both groups showed a statistically nonsignificant difference in postoperative maximum and minimum strabismus angles in every postoperative follow-up visit by the Mann–Whitney test (Figures 4 and 5).

Immediately postoperatively both groups showed 80% satisfactory results. As for the unsatisfactory results, Group A showed 4 cases (16%) with minimum residual ET angle ≥ 10 degrees and one case (4%) with minimum consecutive XT angle ≥ 10 degrees, while those of Group B showed one cases (4%) with minimum residual ET angle ≥ 10 degrees and 4 cases (16%) with minimum consecutive XT angle ≥ 10 degrees.

By the end of the first postoperative month, 2 cases of Group A (8%) with the minimum residual ET angle improved to the satisfactory range, increasing the number of satisfactory cases to be 88%. On the contrary, 2 cases of Group B (8%) one with minimum residual ET angle and another one with minimum consecutive XT angle improved to the satisfactory range, increasing the number of satisfactory cases to be 88%.

Patients whose results were unsatisfactory underwent a second intervention by the end of the postoperative second month. Intervention was in the form of lateral rectus (LR) resection in patients with residual ET and LR recession in patients with consecutive XT. All patients remained within the satisfactory range during the rest of follow-up period.

## 4. Discussion

Variable angle strabismus, like IET, can be treated by different methods. Simple recession of the concerned muscles would correct minimal strabismus angle through reducing muscle *F* only [19]. However, strabismus angle variability remains unchanged. A better way to correct the variable angle strabismus is through reduction of extraocular muscles *r* as well. Two surgical techniques can be used including Faden technique and Y-split recession. Priglinger and Hamenter [17], Haslwanter et al. [18], and Hoerantner et al. [19] have previously shown that the biomechanics underlying these techniques are quite different. In Faden technique, the muscle is sutured to the posterior half of the globe (which is not without complications in high myopic and aphakic eyes) [17–19]. This will change the arc of contact of the muscle with the globe reducing *r* [12]. It would also make the *F* exerted by muscle contraction split into a radial *F* component (pulling at the suture) and a tangential *F* component (rotating the eye). In higher globe excursions, *F* becomes radial leading to blocked ocular motility [18]. With Y-split recession, the muscle is split along 15 mm, and both split-halves are reattached, as shown in Figure 1. Thus, *r* of the muscle is reduced as both halves slide to the side, without any pull in the radial direction due to absence of posterior attachment [1, 20, 21]. It also reduces *F* due to associating recession [19]. It also reduces the difference between larger near deviation and distance deviation with stable long-term effects [22, 23]. Unlike the Faden technique, the *T* reduction with Y-split recession is approximately constant throughout the oculomotor range resulting in less incomitance than that with Faden technique [17–19].

This study is, as far reviewed in the literature, the first study comparing these 2 techniques in this category of patients.

Hoerantner et al. [19] reported that around 1% of the all patients who have undergone Y-split recession have been reoperated a few months later. This was different from the current study which showed that 12% of patients with Y-split recession underwent a second operation. The difference could be attributed to the difference in the number of patients (228 versus 50 patients in the current study). The split muscle was found with no scar tissue between globe and muscle, and the muscle was situated as it was immediately after Y-split surgery. The surface of the muscle halves was covered by a smooth, white tissue unattached to any surrounding structure.

In the current study, the larger *T*   reduction achieved with the de Decker's Faden technique in adduction showed no advantage over Y-split recession which showed equal results to those of the de Decker's Faden technique.

In a study performed by Hoerantner et al. [24], they indicated that Y-split recession represents a powerful alternative to Faden technique. During follow-up visit three months postoperatively, patients with Y-split recession showed a significantly more decreases in maximum strabismus angle. They attributed these improved results to surgical advantages of this technique which reduces *r* and also *F* (through recession) in one operative step, which allows for greater flexibility in the choice of the surgical parameters. However, the recession is influenced by reduced *r* of the operated muscle; therefore, the calculation of the muscle recession at a shorter *r* needs a smaller dose-effect relationship. The postoperative angle reduction within days, respective over longer time, may change by a feedback mechanism and adaptation effect. This was in contrast to the current study which showed similar success rate in both groups. This could be attributed to different Faden technique (Cüppers versus de Decker's in the current study) and different numbers of patients (100 versus 50 patients in the current study). They showed that the surgical area in Y-split recession is anterior to that in the Faden technique and is therefore easier to access, safer, and more accurate [17–19].

In the current study, both techniques showed a significant reduction in both static and dynamic strabismus angles. This was similar to the results obtained by Hoerantner et al. [19] and Hoerantner et al. [24].

## 5. Conclusion

This study shows that both Y-split recession and de Decker's Faden techniques are efficient procedures in 88% of infantile ET. Long-term follow-up will tell if these results are stable with time and if there will be a reduction in the rate of recurring surgery.

## Figures and Tables

**Table 1:** The average values of the maximum strabismus angles preoperatively, one day; one week; one, three, and six months postoperatively.

	Maximum strabismus angle (degrees ± SD)					
	Preoperative	1-day postoperative	1-week postoperative	1-month postoperative	3-month postoperative	6-month postoperative
Y-splitting	25.8 ± 8.124	1.6 ± 6.4096	1.2 ± 6.1712	1.2 ± 6.1712	0	0
		*P* < 0.001	*P* < 0.001	*P* < 0.001	*P* < 0.001	*P* < 0.001
Faden	26.6 ± 7.0297	−0.6 ± 6.69	−1 ± 5.5902	−1 ± 5.5902	0	0
		*P* < 0.001	*P* < 0.001	*P* < 0.001	*P* < 0.001	*P* < 0.001

**Table 2:** The average values of the minimum strabismus angles preoperatively, one day; one week; one, three, and six months postoperatively.

	Minimum (static) strabismus angle (degrees ± SD)					
	Preoperative	1-day postoperative	1-week postoperative	1-month postoperative	3-month postoperative	6-month postoperative
Y-splitting	12 ± 3.2275	1 ± 5	0.6 ± 4.6368	0.2 ± 4.2032	0	0
		*P* < 0.001	*P* < 0.001	*P* < 0.001	*P* < 0.001	*P* < 0.001
Faden	13 ± 3.5355	−0.4 ± 4.5461	−0.8 ± 4	−0.4 ± 3.5119	0	0
		*P* < 0.001	*P* < 0.001	*P* < 0.001	*P* < 0.001	*P* < 0.001

**Table 3:** The average values of the dynamic strabismus angles preoperatively, one day; one week; one, three, and six months postoperatively.

	Dynamic strabismus angle (degrees ± SD)					
	Preoperative	1-day postoperative	1-week postoperative	1-month postoperative	3-month postoperative	6-month postoperative
Y-splitting	13.8 ± 6.172	0.6 ± 2.20	0.6 ± 2.20	0.6 ± 2.20	0	0
		*P* < 0.001	*P* < 0.001	*P* < 0.001	*P* < 0.001	*P* < 0.001
Faden	13.6 ± 4.21	0.6 ± 1.66	0.6 ± 1.66	1.0 ± 2.12	0	0
		*P* < 0.001	*P* < 0.001	*P* < 0.001	*P* < 0.001	*P* < 0.001

## Data Availability

The data used to support the findings of this study are available from the corresponding author upon request.
